# Supplementary material for: A Pro-Inflammatory Stimulus versus Extensive Passaging of DITNC1 Astrocyte Cultures as Models to Study Astrogliosis
Source: Int J Mol Sci. 2024 Aug 30;25(17):9454. doi: 10.3390/ijms25179454 (PMC11394751; doi:10.3390/ijms25179454)
Supplement: Supplementary file 1 [file ijms-25-09454-s001.zip › ijms-3127603-supplementary.pdf]

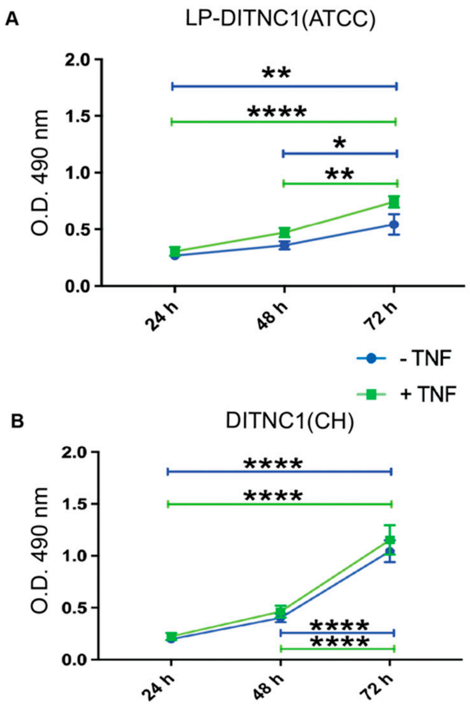

Figure S1: Proliferation of DITNC1 cells with or without TNF. Proliferation was evaluated using the MTS assay in LP DITNC1(ATCC) and in DITNC1(CH) cells treated with TNF (+TNF, green line) or without TNF (-TNF, blue line) after incubating them for 24, 48, and 72 h. Values in the graph indicate the mean of optical density (O.D.) values at 490 nm measured at the different time points (n = 3) (mean ± s.e.m.). *p < 0.05, **p < 0.01, and ****p < 0.001.
